# Supplementary material for: A novel thermoregulatory role for PDE10A in mouse and human adipocytes
Source: EMBO Mol Med. 2016 May 31;8(7):796–812. doi: 10.15252/emmm.201506085 (PMC4931292; doi:10.15252/emmm.201506085)
Supplement: Supplementary file 1 — Appendix [file EMMM-8-796-s001.pdf]

A novel thermoregulatory role for PDE10A in mouse and human adipocytes

Appendix

Table of Contents:

Appendix Figures 12

Appendix Tables 2

Appendix Figure Legends 12

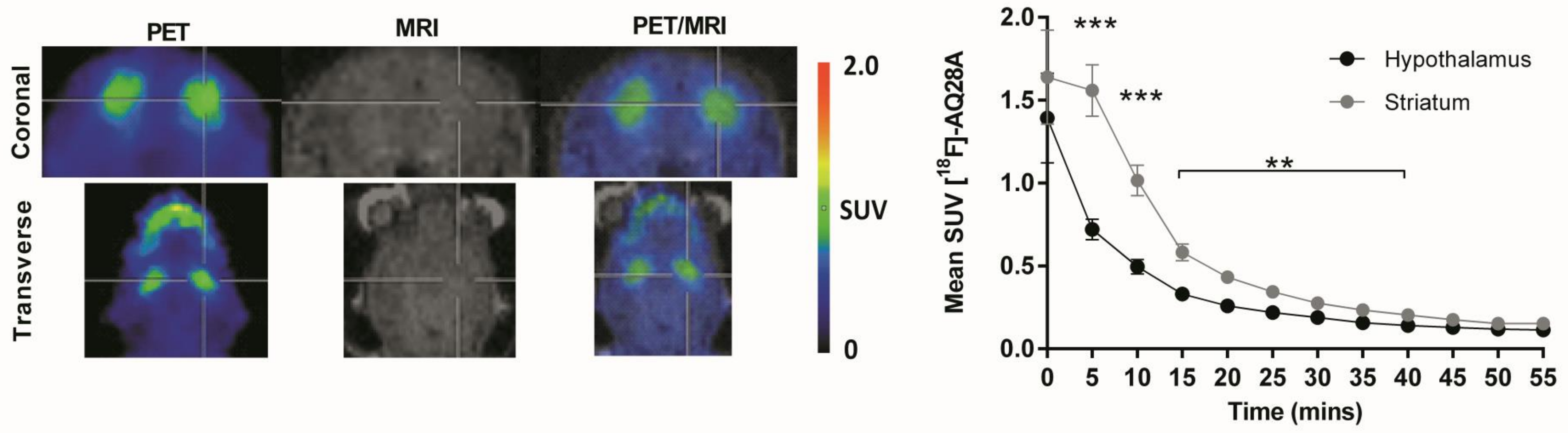

Appendix Figure S1.

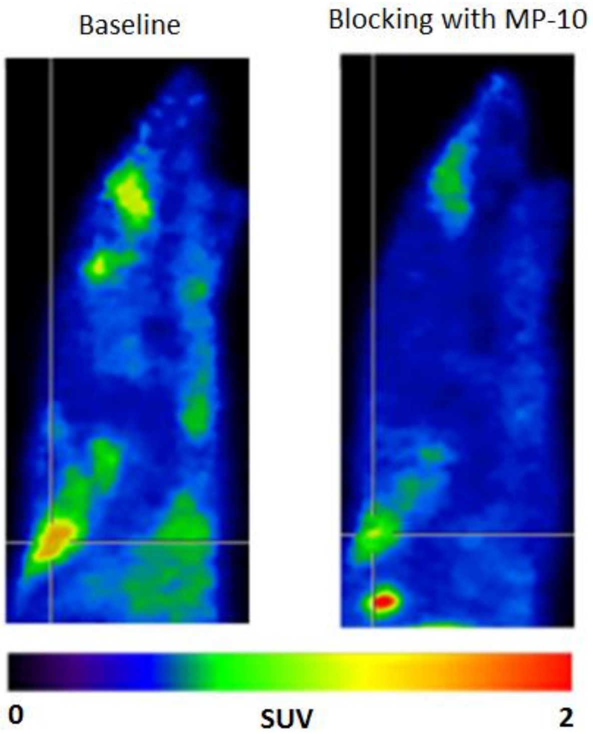

Appendix Figure S2.

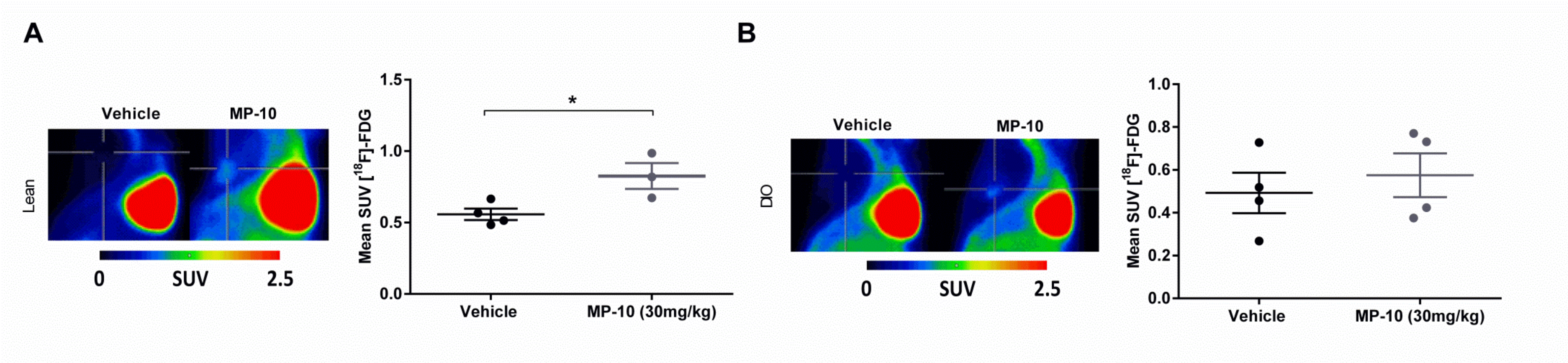

Appendix Figure S3.

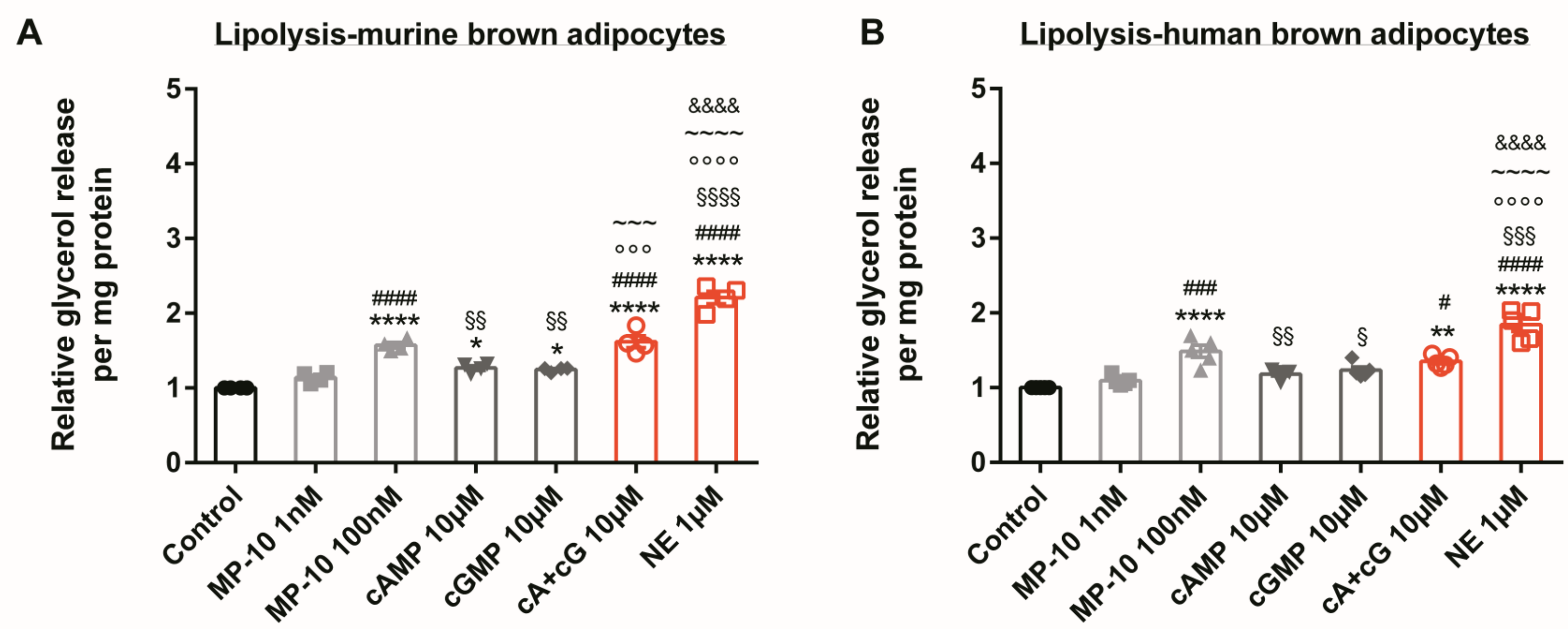

Appendix Figure S4.

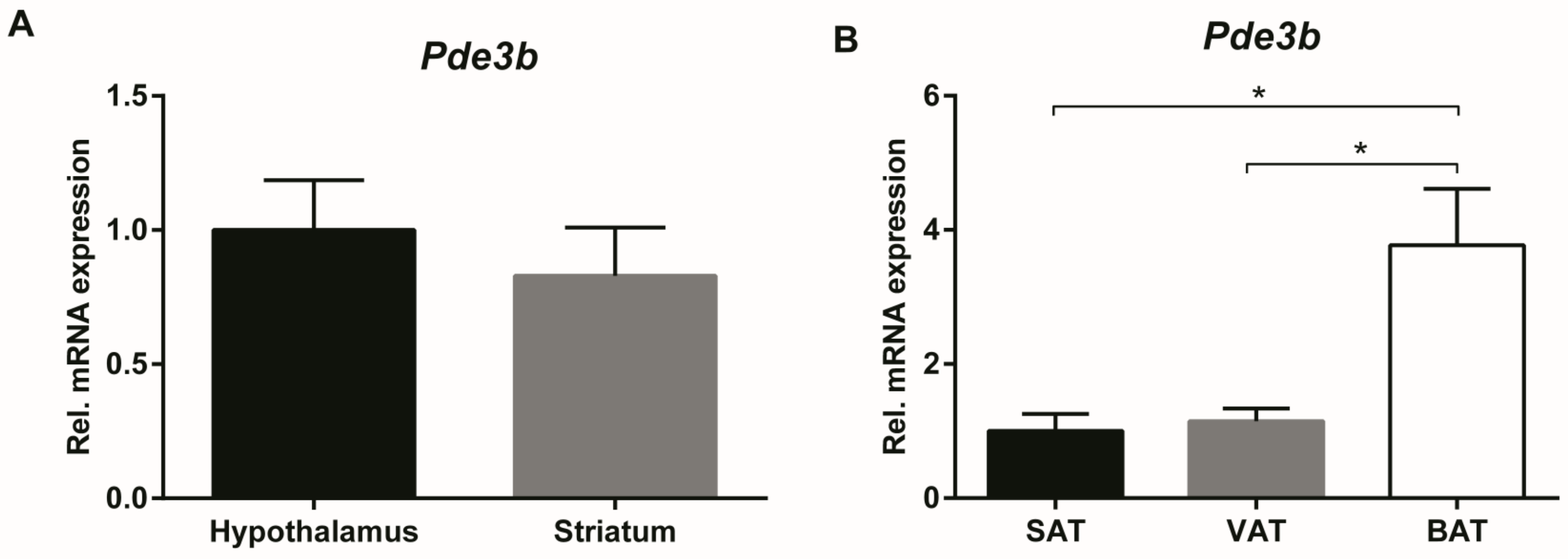

Appendix Figure S5.

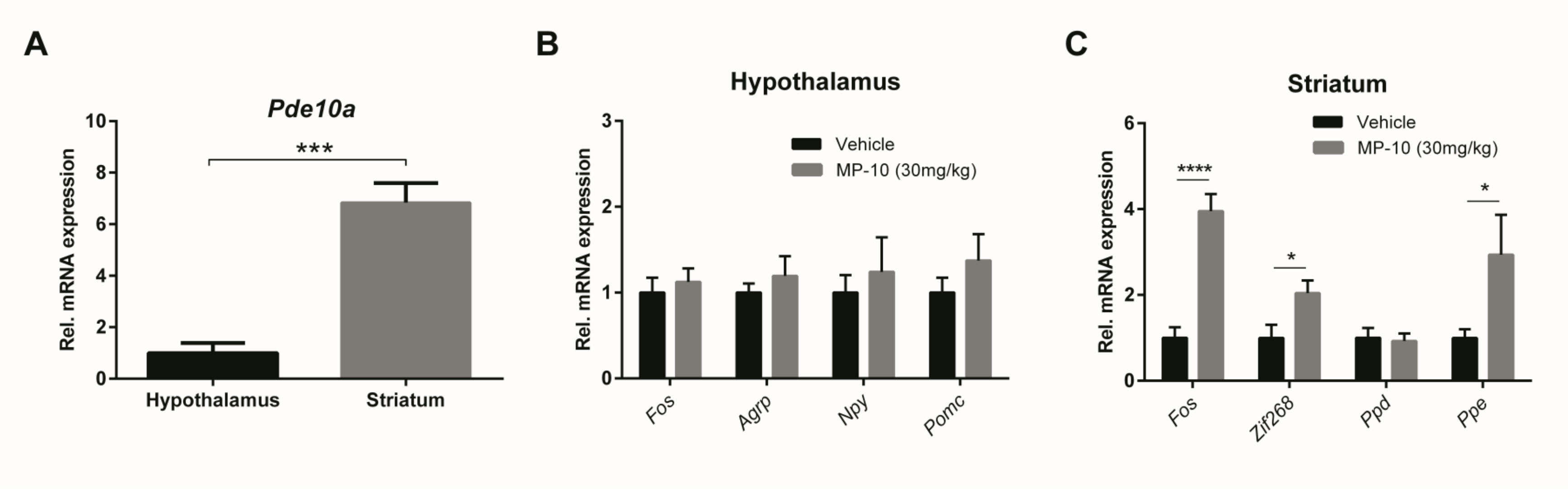

Appendix Figure S6.

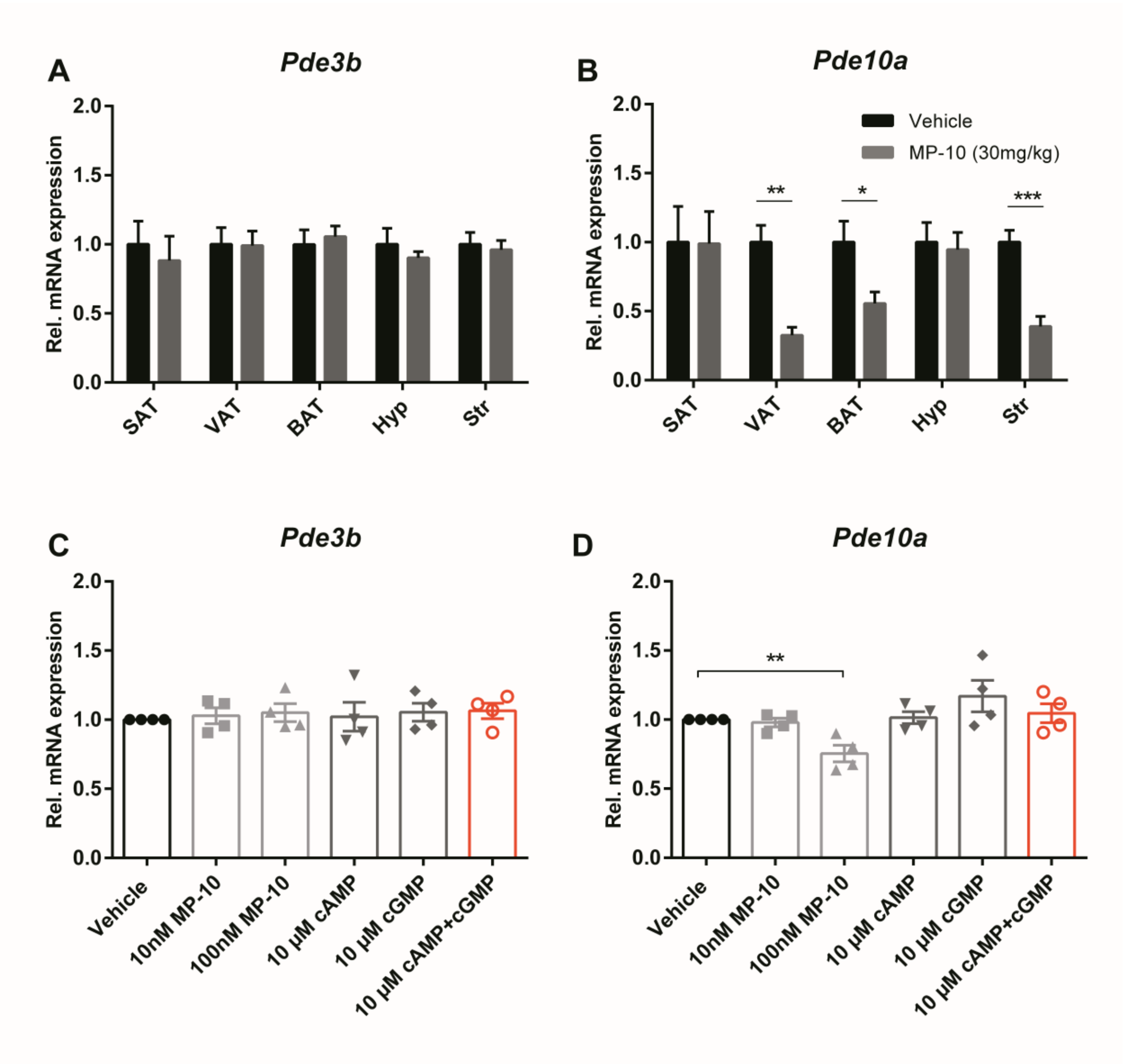

Appendix Figure S7.

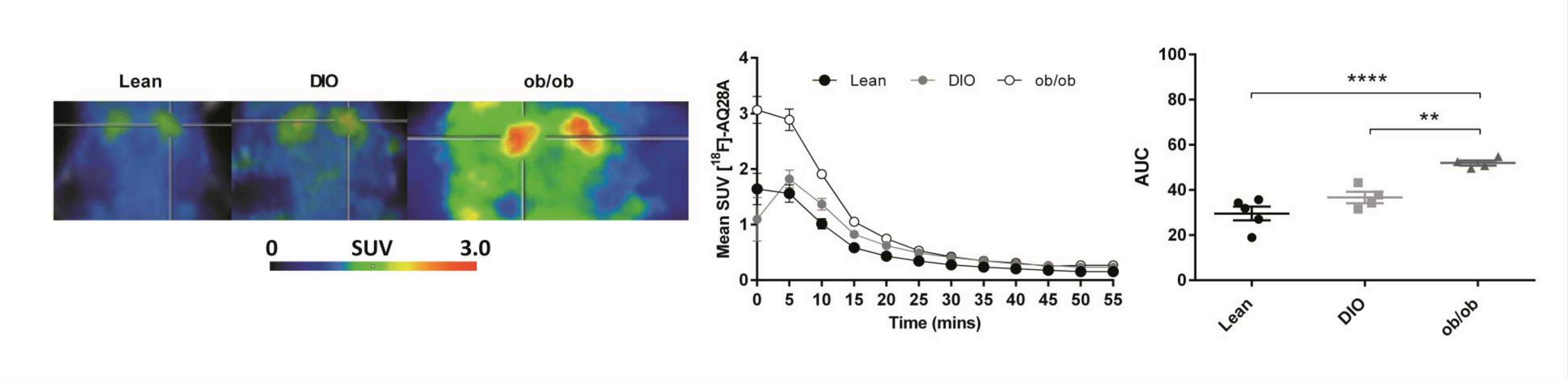

Appendix Figure S8.

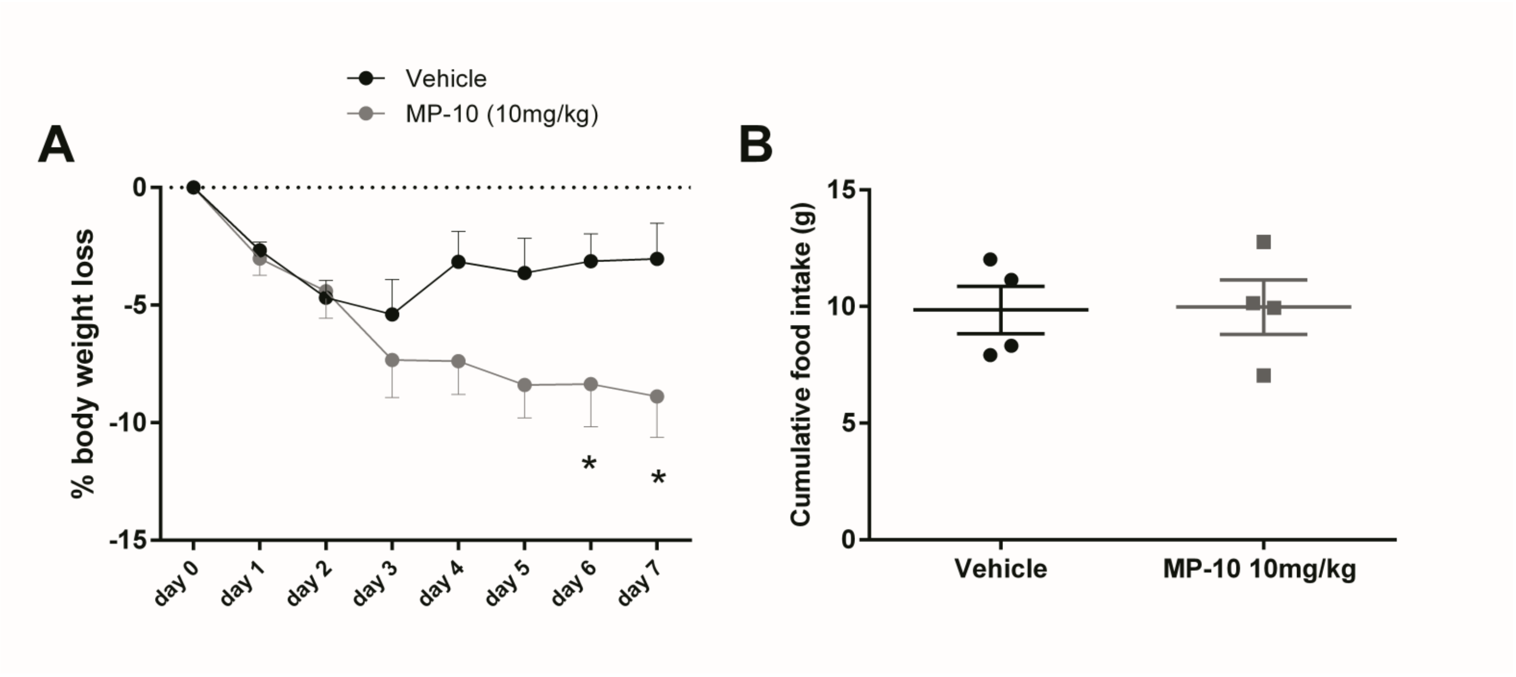

Appendix Figure S9.

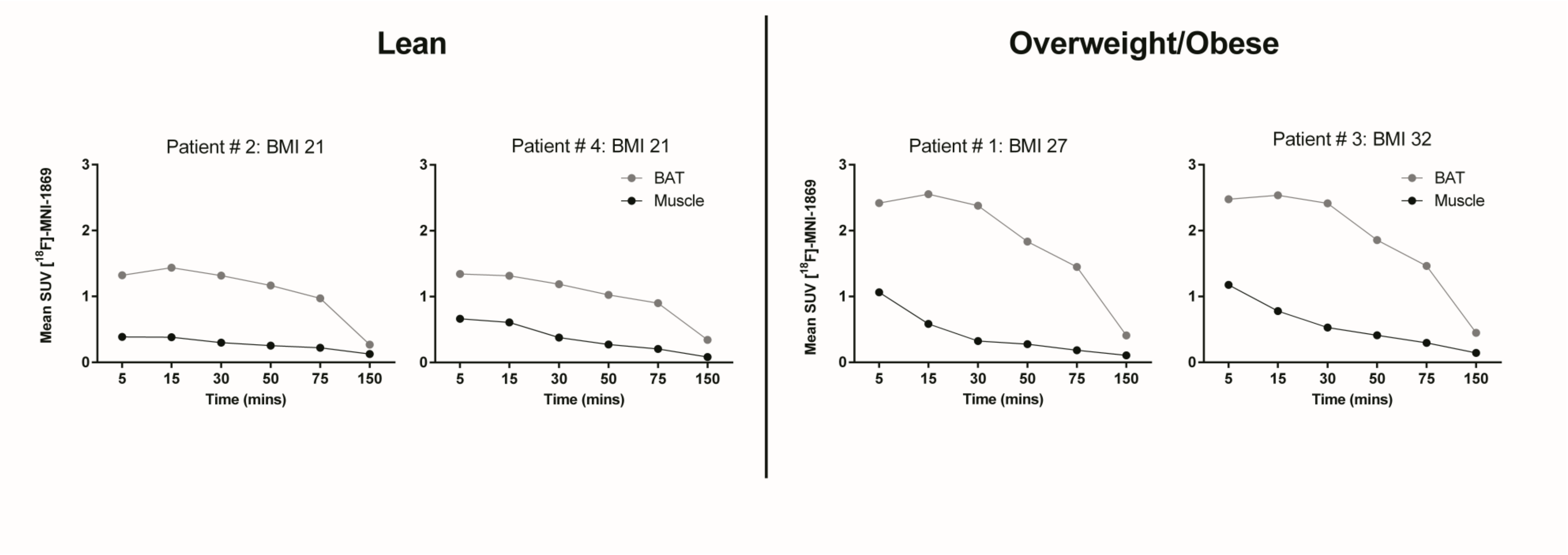

Appendix Figure S10.

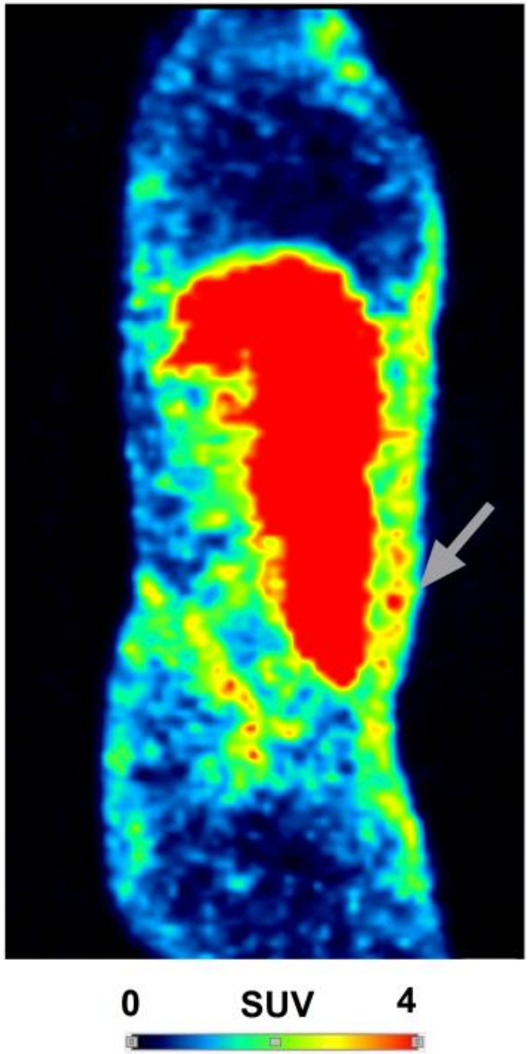

Appendix Figure S11.

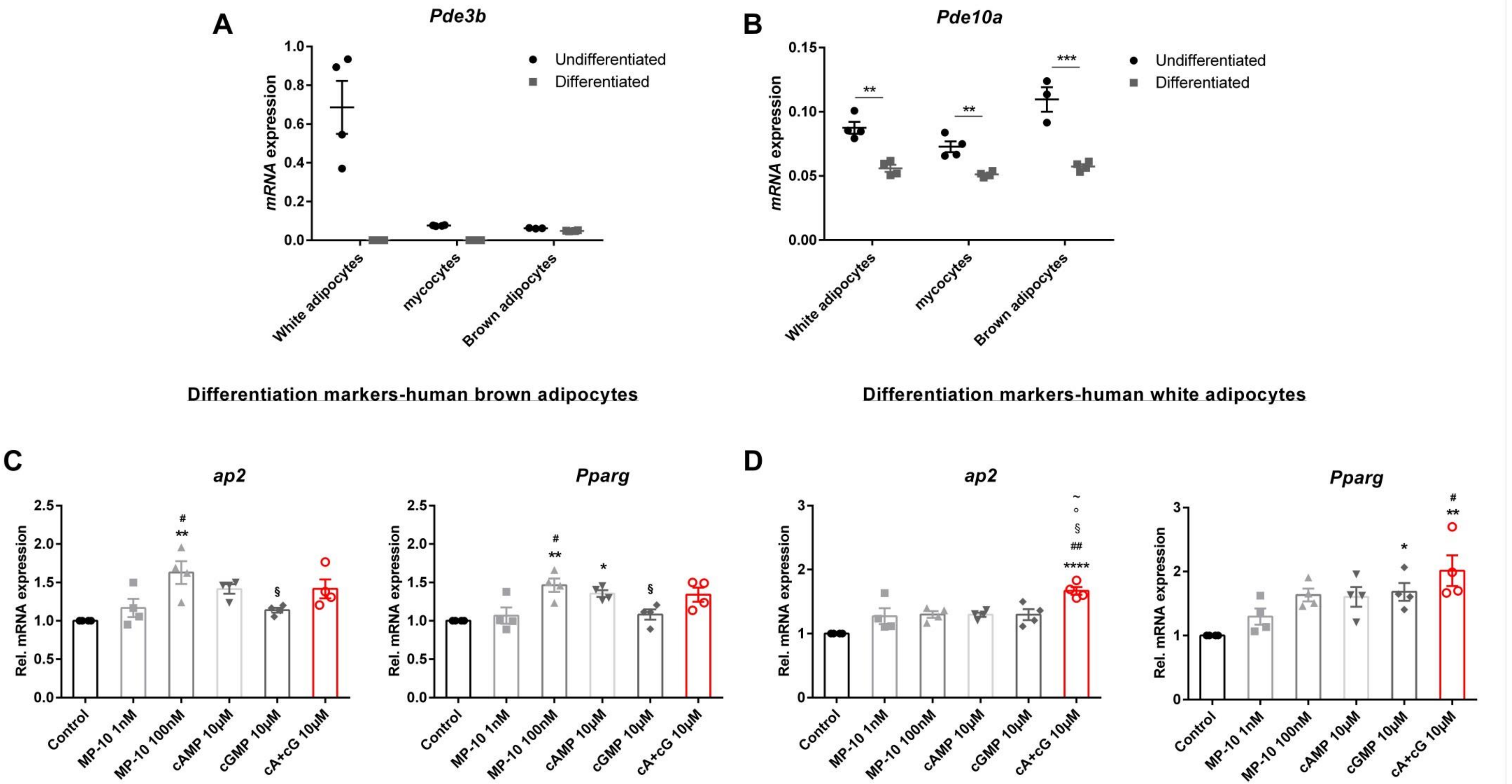

Appendix Figure S12.

**Mouse Genes**	**Forward (5’-3’)**	**Reverse (5’-3’)**
Agrp	GGC ACA AGA GAC CAG GAC AT	ACT TCT TCT GCT CGG TCT GC
Beta actin	CAT TGC TGA CAG GAT GCA GA	CTG ATC CAC ATC TGC TGG AA
Cidea	TGC TCT TCT GTA TCG CCC AGT	GCC GTG TTA AGG AAT CTG CTG
Fos	AAA CCG CAT GGA GTG TGT TGT TCC	TCA GAC CAC CTC GAC AAT GCA TGA
Npy	AGA GAT CCA GCC CTG AGA CA	TTT CAT TTC CCA TCA CCA CA
Pde3b	ACG GAA ACC AAA GCA GAT TC	GCA GCC ATA ACT CAT ATC TGG A
Pde10a	TAC CAG ACA GGG TCG CTG A	TGG CCA TAG TTT GGT CAC AG
Pgc1alpha	AGC CGT GAC CAC TGA CAA CGAG	GCT GCA TGG TTC TGA GTG CTAAG
Pomc	GAA CAG CCC CTG ACT GAA AA	AAC GTT GGG GTA CAC CTT CA
Ppd	GTG CAG TGA GGA TTC AGG ATG GG	GAG CTT GGC TAG TGC ACT GTA GC
Ppe	CTA AAT GCA CGT ACC GCC TGG TT	CGA TGT TAT CCC AAG GGA ACT CG
Prdm16	ACA GGC AGG CTA AGA ACC AG	CGT GGA GAG GAG TGT CTT CAG
Ucp1	AGG GTT TGT GGC TTC TTT TC	TGG TTG GTT TTA TTC GTG GT
Tbx1	GGC AGG CAG ACG AAT GTT C	TTG TCA TCT ACG GGC ACA AAG
Tmem26	ACC CTG TCA TCC CAC AGA G	TGT TTG GTG GAG TCC TAA GGT C
Zif268	TCG GCT CCT TTC CTC ACT CA	CTC ATA GGG TTG TTC GCT CGG
**Human Genes**	**Forward (5’-3’)**	**Reverse (5’-3’)**
Cidea	GGC AGG TTC ACG TGT GGA TA	GAA ACA CAG TGT TTG GCT CAA GA
Dio2	GT CAC TGG TC AGC GTG GTT TT	TTC TTC ACA TCC CCC AAT CCT
Gapdh	TGG TCT CCT CTG ACT TCA	GTG AGG GTC TCT CTC TTT CCT
Pgc1alpha	CTG TGT CAC CAC CCA AAT CCT TAT	TGT GTC GAG AAA AGG ACC TTG A
Prdm16	GAA ACT TTA TTG CCA ATA GTG AGA TGA	CCG TCC ACG ATC TGC ATG T
Ucp1	GGA ACA ATC ACC GCT GTG GT	ATC CTG AGA GAG GCG CAG CT
Pde10a	TGA GAA AGG AAT TGC TGG CCA AGT	TCA TGA CAC AGA CAC GCA ATC AG
Pde3b	CCT TCT TCT TCC TCA CCT GCT T	GAT CAA CTC CAT TTC CAC CTC C
Ap2	TGT GCA GAA ATG GGA TGG AAA	CAA CGT CCC TTG GCT TAT GCT
Ppargamma	AGC CTC ATG AAG AGC CTT CCA	TCC GGA AGA AAC CCT TGCA

Appendix Table S1.

Sequences for primers used in RT-qPCR reactions

	**Age (years) at time of scan**	**Gender**	**BMI (kg/m^2)**	**Race**	**Ethnicity**
**Patient # 1**	42	Male	27	White	Hisp
**Patient # 2**	48	Female	21	White	Non-Hisp
**Patient # 3**	40	Male	32	White	Non-Hisp
**Patient # 4**	35	Female	21	White	Non-Hisp

Appendix Table S2.

Patient demographics for retrospective PET scan analysis

Appendix Figure S1.

Selective uptake of [^{18}F]-AQ28A in striatum of mice

Representative PET, MRI, and fused PET/MRI images of the brain of a mouse that received the PDE10A radioligand [^{18}F]-AQ28A. Striatum is highlighted in each image by the crosshairs. The mean standardized uptake value of [^{18}F]-AQ28A in striatum and hypothalamus throughout the dynamic was calculated and compared for each separate animal (n = 5). ****** P*** = 0.000537 (5 mins), ****** P*** = 0.000461 (10 mins), ***** P*** = 0.00109 (15 mins), ***** P*** = 0.00299 (20 mins), ***** P*** = 0.00487 (25 mins), ***** P*** = 0.00171 (30 mins), ***** P*** = 0.00100 (35 mins) and ***** P*** = 0.00103 (50mins) using unpaired 2-tailed Student's *t* tests.

Data information: Data are represented as mean +/- SEM.

Appendix Figure S2.

[^{18}F]-AQ28A is displaced by the selective PDE10A inhibitor MP-10

Representative PET images of a sagittal view of the upper body of a mouse that received the PDE10A radioligand [18F]-AQ28A without (left panel) and 20 minutes after (right panel) pre-treatment with MP-10 (5mg/kg). BAT is highlighted in each image by the crosshairs.

Appendix Figure S3.

Stimulation of *in vivo* glucose uptake by BAT in response to acute pharmacological inhibition of PDE10A with MP-10 is blunted in diet induced mice

A. Representative PET images of a sagittal view of the thoracic region of *ad libitum* fed lean mice treated with either MP-10 (30mg/kg) (n = 4) or vehicle (n = 3) prior to receiving [^{18}F]-FDG. Interscapular brown adipose tissue (BAT) in each image is highlighted by the crosshairs. The mean standardized uptake value (SUV) of [^{18}F]-FDG in BAT after both treatments was calculated and compared between groups. **** P*** = 0.0299 using unpaired 2-tailed Student's *t* test.

B. Representative PET images of a sagittal view of the thoracic region of *ad libitum* fed diet induced obese (DIO) mice treated with either MP-10 (30mg/kg) (n = 4) or vehicle (n = 4)prior to receiving [^{18}F]-FDG. Interscapular brown adipose tissue (BAT) in each image is highlighted by the crosshairs.

Data information: Individual data are shown where bars represent mean +/- SEM.

Appendix Figure S4.

Inhibition of PDE10A with MP-10 stimulates lipolysis in mouse and human brown adipocytes

A. Relative glycerol release in media of cultured mouse brown adipocytes in response to acute treatment (2 hours) with DMSO (control), MP-10 (1nM and 100nM), cyclic AMP (cAMP – 10µM), cyclic GMP (cGMP - 10µM), cAMP and cGMP (cA + cG – 10µM) and norepinephrine (NE – 1µM) (n = 4 separate cultures). **** *P* < 0.0001 (MP-10 100nM vs control), #### *P* < 0.0001 (MP-10 1nM vs MP-10 100nM), * *P* = 0.0127 (cAMP 10µM vs control), §§ *P* = 0.0036 (cAMP 10µM vs MP-10 100nM), * *P* = 0.0232 (cGMP 10µM vs control), §§ *P* = 0.0019 (cGMP 10µM vs MP-10 100nM), **** *P* < 0.0001 (cA + cG 10µM vs control), #### *P* < 0.0001 (cA + cG 10µM vs MP-10 1nM), °°° *P* = 0.0008 (cA + cG 10µM vs cAMP 10µM), ~~~ *P* = 0.004 (cA + cG 10µM vs cGMP 10 µM), **** *P* < 0.0001 (NE 1µM vs control, #### *P* < 0.0001 (NE 1µM vs MP-10 1nM), §§§ *P* = 0.0009 (NE 1µM vs MP-10 100nM), °°°° *P* < 0.0001 (NE 1µM vs cAMP 10µM), ~~~~ *P* < 0.0001 (NE 1µM vs cGMP 10 µM) and &&&& *P* < 0.0001 (NE 1µM vs cA + cG 10µM) using one-way analysis of variance (ANOVA) with Tukey's post-hoc test.

B. Relative glycerol release in media of cultured primary human brown adipocytes in response to acute treatment (4 hours) with DMSO (control), MP-10 (1nM and 100nM), cyclic AMP (cAMP – 10µM), cyclic GMP (cGMP - 10µM), cAMP and cGMP (cA + cG – 10µM) and norepinephrine (NE – 1µM) (n = 5 separate cultures). **** *P* < 0.0001 (MP-10 100nM vs control), ### *P* = 0.0002 (MP-10 1nM vs MP-10 100nM), §§ *P* = 0.0056 (cAMP 10µM vs MP-10 100nM), § *P* = 0.0301 (cGMP 10µM vs MP-10 100nM), ** *P* = 0.0011 (cA + cG 10µM vs control), # *P* = 0.0254 (cA + cG 10µM vs MP-10 1nM), **** *P* < 0.0001 (NE 1µM vs control), #### *P* < 0.0001 (NE 1µM vs MP-10 1nM), §§§§ *P* < 0.0001 (NE 1µM vs MP-10 100nM), °°°° *P* < 0.0001 (NE 1µM vs cAMP 10µM), ~~~~ *P* < 0.0001 (NE 1µM vs cGMP 10 µM) and &&&& *P* < 0.0001 (NE 1µM vs cA + cG 10µM) using one-way analysis of variance (ANOVA) with Tukey's post-hoc test.

Data information: Individual data are shown where bars represent mean +/- SEM.

Appendix Figure S5.

Relative *Pde3b* mRNA expression in different brain regions and fat depots

A, B RT-qPCR analysis of relative *Pde3b* mRNA expression in hypothalamus and striatum and inguinal subcutaneous white adipose tissue (SAT), peri-ovarian visceral white adipose tissue (VAT), and interscapular brown adipose tissue (BAT) for each separate animal (n = 5). * *P* = 0.0102 (SAT vs BAT) and * *P* = 0.0123 (VAT vs BAT) using unpaired 2-tailed Student's *t* tests.

Data information: Data are represented as mean +/- SEM.

Appendix Figure S6.

Acute pharmacological inhibition of PDE10A regulates gene expression in striatum, but not hypothalamus

A. RT-qPCR analysis of relative mRNA expression of *Pde10a* in hypothalamus and striatum for each separate animal (n = 5). ****** P*** = 0.0004 using unpaired 2-tailed Student's *t* test.

B, C RT-qPCR analysis of relative mRNA expression of candidate genes in hypothalamus and striatum after treatment of lean mice with MP-10 (30 mg/kg) (n=7) or vehicle control (n = 6). ******* P*** < 0.0001 (*Fos* in striatum), **** P*** = 0.0338 (*Zif268*) and **** P*** = 0.0374 (*Ppe)* using unpaired 2-tailed Student's *t* tests.

Data information: Data are represented as mean +/- SEM.

Appendix Figure S7.

Pharmacological inhibition of PDE10A with MP-10 decreases mRNA expression of *Pde10a,* but not *Pde3b*

A, B RT-qPCR analysis of relative mRNA expression of *Pde3b* and *Pde10a* in inguinal subcutaneous white adipose tissue (SAT), peri-ovarian visceral white adipose tissue (VAT), interscapular brown adipose tissue (BAT), hypothalamus (Hyp) and striatum (Str) after treatment of lean mice with MP-10 (30 mg/kg) (n=7) or vehicle (n = 6). ***** P*** = 0.0077 (*Pde10a* in VAT), ****P*** = 0.0251 (*Pde10a* in BAT) and ******P*** = 0.0005 (*Pde10a* in Str) using unpaired 2-tailed Student's *t* tests.

C, D RT-qPCR analysis of relative mRNA expression of *Pde3b* and *Pde10a* in primary human brown adipocytes in response to chronic treatment (8 hours) with DMSO control, MP-10 (1nM and 100nM), cyclic AMP (cAMP – 10µM), cyclic GMP (cGMP - 10µM) and cAMP and cGMP (10µM) (n = 4 separate cultures). ***** P*** = 0.0064 (*Pde10a* with 100nM MP-10) using unpaired 2-tailed Student's *t* test.

Data information: Data are represented as mean +/- SEM. Graphs in **C.** and **D.** show data from separate experiments where bars represent mean +/- SEM.

Appendix Figure S8.

Expression of PDE10A in striatum is increased is increased in different mouse models of obesity

Representative fused PET/MRI images of the transverse view of the brains of lean, diet induced obese (DIO) and leptin deficient (ob/ob) mice that received the PDE10A radioligand [^{18}F]-AQ28A. Striatum is highlighted in each image by the crosshairs. The mean standardized uptake value of [^{18}F]-AQ28A in striatum throughout the dynamic scan was calculated in lean (n = 5), DIO (n = 4) and ob/ob mice (n=4) with associated area under the curve (AUC). ******* P*** = 0.0001 (lean vs ob/ob) and ***** P*** = 0.0015 (DIO vs ob/ob) using unpaired 2-tailed Student's *t* tests.

Data information: Data are represented as mean +/- SEM. Graph in right panel shows individual data where bars represent mean +/- SEM

Appendix Figure S9.

Chronic inhibition of PDE10A with MP-10 causes weight loss without affecting food intake in diet induced obese C57BL/6 mice

A, B The percentage (%) change in body weight and cumulative food intake of diet induced obese (DIO) mice in response to daily treatment with MP-10 (10mg/kg) (n = 4) or vehicle (n = 4) for a week. * ***P*** = 0.05 (day 6) and * ***P*** = 0.05 (day 7) using two way analysis of variance (ANOVA) with Sidak's post-hoc test.

Data information: * ***P*** < 0.05 using Two-Way analysis of variance (ANOVA). Data are represented as mean +/- SEM. Graph in B. shows individual data where bars represent mean +/- SEM

Appendix Figure S10.

Expression of PDE10A in supraclavicular BAT of humans increases with BMI

The mean standardized uptake value (SUV) of [^{18}F]-MNI-1869 in supraclavicular BAT and skeletal muscle was calculated for individual patients with BMI indicated.

Appendix Figure S11.

Uptake of [^{18}F]-MNI-1869 by subcutaneous white adipose tissue of humans

Representative PET image of the upper body of a human subject who received the PDE10A radioligand [^{18}F]-MNI-1869. Subcutaneous abdominal white adipose tissue (SAT) is highlighted by the grey arrow.

Appendix Figure S12.

PDE10A is expressed in human brown preadipocytes and its pharmacological inhibition with MP-10 promotes brown adipocyte differentiation

A, B RT-qPCR analysis of mRNA expression of *Pde3b* and *Pde10a* in primary human white adipocyte (n = 4 separate cultures), myocyte (n = 4 separate cultures) and brown adipocyte (n = 3 separate cultures) precursor and mature cells (n = 4 separate cultures). ***** P*** = 0.0011 (undifferentiated vs differentiated white adipocytes), ***** P*** = 0.0025 (undifferentiated vs differentiated myocytes) and ****** P*** = 0.0025 (undifferentiated vs differentiated brown adipocytes) using unpaired 2-tailed Student's *t* tests.

C. RT-qPCR analysis of relative mRNA expression of the adipogeneic markers ap2 and Pparg in primary human brown adipocyte precursors in response to treatment every other day during differentation with DMSO (control), MP-10 (1nM and 100nM), cyclic AMP (cAMP – 10µM), cyclic GMP (cGMP - 10µM) and cAMP and cGMP (10µM) (n = 4 separate cultures). $^{\#\#}$ ***P*** = 0.0026 (MP-10 100nM vs control), $^{\#}$ ***P*** = 0.0336 (MP-10 100nM vs MP-10 1nM), § ***P*** = 0.0218 (MP-10 100nM vs cGMP 10µM) using one-way analysis of variance (ANOVA) with Tukey's post-hoc test.

D. RT-qPCR analysis of relative mRNA expression of the adipogeneic markers ap2 and Pparg in primary white adipocyte precursors in response to treatment every other day during differention with DMSO (control), MP-10 (1nM and 100nM), cyclic AMP (cAMP – 10µM), cyclic GMP (cGMP - 10µM) and cAMP and cGMP (10µM) (n = 4 per group). ******* P*** < 0.0001 (cA + cG 10µM vs control), $^{\#\#}$ ***P*** = 0.0099 (cA + cG 10µM vs MP-10 1nM), § ***P*** = 0.0175, **°** ***P*** = 0.0161 (cA + cG 10µM vs cAMP 10µM), **~** ***P*** = 0.0161 (cA + cG 10µM vs cGMP 10 µM) using one-way analysis of variance (ANOVA) with Tukey's post-hoc test.

Data information: Individual data from separate experiments are shown where bars represent mean +/- SEM.
